# Small mammal herbivores mediate the effects of soil nitrogen and invertebrate herbivores on grassland diversity

**DOI:** 10.1002/ece3.4991

**Published:** 2019-02-21

**Authors:** Nicole Poe, Katharine L. Stuble, Lara Souza

**Affiliations:** ^1^ Oklahoma Biological Survey and Microbiology and Plant Biology Department University of Oklahoma Norman Oklahoma; ^2^ The Holden Arboretum Kirtland Ohio

**Keywords:** aboveground biomass, compositional similarity, grassland, herbivores, invertebrates, plant diversity, plant evenness, plant richness, small mammals, soil nitrogen

## Abstract

1. Simultaneous reductions in herbivore abundance and increases in nitrogen deposition have led to radical shifts in plant communities worldwide. While the individual impacts of these human‐caused disturbances are apparent, few studies manipulate both herbivory and N, nor differentiate among herbivore guilds, to understand contingencies in the ability of these drivers to affect producer diversity and productivity. As such, understanding how the main and combined effects of increasing soil N with declining herbivores may influence plant community structure and function is critical to better understand the future of grassland ecosystems under multiple global change drivers.
2. In this study, we asked: (a) What are the main effects of small mammal herbivores, invertebrate herbivores, and soil N on plant community structure and function? and (b) Are the effects of invertebrate herbivores and soil N on plant community structure and function contingent on small mammal herbivory? We used a nested design, with invertebrate and soil N treatments nested within small mammal manipulations in an existing tallgrass prairie. We measured plant community structure by quantifying plant richness, evenness, diversity, and composition across two full growing seasons. We also recorded total aboveground biomass to quantify grassland productivity.
3. We found that small mammal herbivores strongly shaped plant diversity, species composition, and productivity. Small mammal herbivores also mediated the effects of soil N and invertebrate herbivores on grassland community structure, but not composition or productivity. Small mammal reduction lowered plant species richness while increasing aboveground biomass and altering compositional similarity. Invertebrate herbivores, in the presence of small mammals, promoted plant dominance by reducing evenness without altering compositional similarity. Additionally, soil nitrogen addition reduced plant richness, but only when small mammals were reduced, and no effects were detected on compositional similarity or productivity.
4. Our findings provide further evidence that temperate grasslands can be strongly influenced by consumers, and that consumers mediate the effects of resources as well as other consumer guilds on producer evenness and richness. Taken together, we provide evidence of strong contingencies in the drivers of grassland structure, with small mammals directly altering plant diversity as well as mediating the effects of soil nitrogen and invertebrate herbivory on plant richness and evenness. Therefore, we suggest it is imperative to consider how consumer guilds and resource types may interact to shape grassland plant communities.

## 1 INTRODUCTION

Herbivore communities and nutrient availability are changing concurrently worldwide (Crain, Kroeker, & Halpern, 2008; Wilcove, Rothstein, Dubow, Phillips, & Losos, 1998). Furthermore, human activity accounts for much of the change in herbivory and nutrient addition. For instance, humans have decreased grazing intensity through management practices (Hughes, 1994; Welch & Scott, 1995). In grasslands specifically, natural disease and population control efforts have caused herbivore populations to decrease (Finch, 2005; Knowles, 2002). Concurrent with this decline in consumers has been an increase in nitrogen (N) inputs (Gruner et al., 2008). Nitrogen inputs have increased more than twofold over preindustrial levels (Galloway et al., 2003; Jefferies & Maron, 1997) due to anthropogenic N deposition from ammonia production and fossil fuel combustion (Galloway et al., 2003), and, most significantly, fertilization (Liu et al., 2013; Nehring, 2016). As a result, these changes in herbivory (top‐down) and soil nutrients (bottom‐up) are altering the structure and function of plant communities.

The effect of herbivory on productivity and diversity within plant communities is variable. Recent studies have found that herbivores can have positive or sometimes neutral effects on plant productivity (Borer, Seabloom, Gruner et al., 2014a; Borer, Seabloom, Mitchell, & Cronin, 2014b; Gruner et al., 2008; Maron & Crone, 2006; Olofsson, de Mazancourt, & Crawley, 2007). Furthermore, the effects of herbivores on plant community diversity differ across productivity gradients, with herbivores promoting diversity under high productivity while the opposite is true in low‐productivity environments (Bakker, Ritchie, Olff, Milchunas, & Knops, 2006; Hillebrand et al., 2007). Additionally, herbivore guilds differ based on feeding patterns, metabolic efficiency, spatial distribution, and size, ultimately leading to variable impacts on the plant community (Gruner et al., 2008; Oduor, Gomez, & Strauss, 2010; La Pierre, Joern, & Smith, 2015; Shurin & Seabloom, 2005). Small mammals play a strong role in structuring grassland ecosystems by removing 30%–70% of aboveground plant biomass while selectively foraging on species with high tissue quality, ultimately altering plant diversity and overall species composition (Howe et al. 2002; Howe, Zorn‐Arnold, Sullivan, & Brown, 2006; Peters, 2007). Similarly, invertebrate herbivores can significantly reduce plant productivity (Carson & Root, 2000; Del‐Val & Crawley, 2005, Gao, Wang, Ba, Bai, & Liu, 2008) while simultaneously altering plant diversity and species composition (Souza, Zelikova, & Sanders, 2015). Even so, few studies have experimentally tested the relative and combined influence of vertebrate and invertebrate herbivore guilds on grassland plant communities.

In addition to the direct and interactive impacts of herbivore guilds, few studies have looked at the interactions between herbivores and soil N on plant diversity and aboveground biomass. A meta‐analysis by Gruner et al. (2008) showed minimal interactive effects of nutrient fertilization and herbivory on producer productivity. Hillebrand et al. (2007), on the other hand, suggested that herbivore and nutrient effects on diversity metrics may be context dependent, with positive effects when producer productivity is high and producer evenness is low, yet negative effects when productivity is low and evenness is high. However, we lack a clear understanding of how soil nutrients interact with distinct herbivore guilds (i.e., small mammal herbivores and invertebrates) to alter plant community structure and function.

To better understand how herbivory and resource availability interact to alter grassland ecosystem structure and function, we manipulated invertebrate herbivory and soil N within an existing small mammal manipulation. We asked the following questions: (a) What are the main effects of declining small mammal herbivore abundances on grassland structure (diversity, richness, evenness, and composition) and function (aboveground biomass)? (b) How does the decline of small mammals influence the relative impacts of invertebrate herbivores and soil N on plant community structure and function? We predicted that: (a) With decreased abundances of small mammals, aboveground plant biomass would increase while diversity would decrease, leading to a shift in plant community composition. We expected this increase in plant biomass concurrent with the reduction of small mammals as a result of a reduction in herbivore consumption of warm‐season grasses (C4 photosynthetic pathway). These grasses can competitively reduce the abundance of forbs (C3 photosynthetic pathway), ultimately reducing overall plant diversity in temperate prairie ecosystems. (b) With a decreased small mammal population, invertebrate herbivore decline would further promote aboveground biomass while reducing plant diversity by favoring competitively dominant warm‐season graminoid species. (c) Nitrogen addition would further promote aboveground biomass, while maintaining plant diversity when small mammals and invertebrate herbivores were reduced. This is because increases in soil nitrogen promotes C3‐forbs that are otherwise outcompeted by warm‐season graminoids in temperate prairie ecosystems; forbs which tend to be reduced in the presence of herbivores.

## 2 MATERIALS AND METHODS

### 2.1 Study site

We conducted our study at Kessler Atmospheric and Ecological Field Station (KAEFS, 34°59′N, 97°31′W), a mixed grass prairie in central Oklahoma, USA. The KAEFS landscape and management practices are representative of Oklahoma's vegetation physiognomy (mixed grassland, riparian, and woody habitats) and grazing regimes. Average annual precipitation is 930 mm, and the mean annual temperature is 16°C, ranging from 3.5°C in January to 27.8°C in July (averaged from 1971 to 2010, data from Oklahoma Climatological Survey). Soils have been characterized as a silt loam (35.3% sand, 55.0% silt, and 9.7% clay) (Zhou, Wan, & Luo, 2007). The most commonly occurring plant species at the study site include the following: *Tridens flavus*, *Bromus racemosus, Commelina communis*, *Andropogon gerardii*, *Crouton glandulosus*, *Dicanthelium oligosanthes*, *Vicia americana*, and *Artemesia ludociviana*. We also identified over 75 other subordinate and transient species, both herbaceous and woody.

### 2.2 Experimental design

We used a nested plot design to address how soil N addition and invertebrate herbivory effects on plant communities may be mediated by small mammal herbivores. We completely randomized invertebrate herbivory and soil N manipulations within existing small mammal reduction and access plots (Supporting Information Appendix S1). We created eight unique treatments which we replicated four times each creating 32 plots total. Previous to the invertebrate and nutrient treatments, four small mammal reduction plots (approximately 7 m × 20 m each) were established (ca. 2006, *Personal Communication*), spanning a total area of 15 m × 40 m. Reduction plots were composed of aluminum flashing (36 cm width) buried 40 cm below the soil surface and galvanized hardware cloth (122 cm width, 0.64 cm mesh) 82 cm above the ground. Adjacent to exclosures, an additional 15 m × 40 m area with no above or buried metal fencing was designated the small mammal access area. Welded wire fencing surrounding the entire site prevented access to all plots by grazing cattle, but did not hinder the movement of small animals. Small mammals were trapped by Sam Noble Natural History Museum mammalogists for three consecutive nights in 2014. They used Sherman live traps (H. B. Sherman Traps, Inc., Tallahassee, Florida) to estimate small mammal abundance following guidelines of the American Society of Mammalogists for animal care and use (Gannon, Sikes, & Comm, 2007). Total small mammal abundance (e.g., small mammal number) was 20% higher in the small mammal access plots relative to the reduction plots, but the average body mass was 80% greater for mammals in the small mammal access plots than exclosures, resulting from a shift to smaller‐bodied species in the reduction plots (Supporting Information Appendix S2). The most common small mammals across access and reduction plots were the white‐footed mouse (*Peromyscus leucopus*), cotton rat (*Sigmondon* spp.), and woodland vole (*Microtus pinetorum*). These species are commonly known to consume plant material (Cameron & Spencer, 1981; Kurta, 1995; Lackey, Huckaby, & Ormiston, 1985).

In the summer of 2013, we established invertebrate herbivore manipulation treatments nested within the existing small mammal herbivore removal experiment. We had two invertebrate removal treatments: (a) “invertebrate reduction,” consisting of an aboveground mesh exclosure with no invertebrate access (a 1.1 m in diameter × 1.5 m tall metal cage enclosed in mesh (C18A mesh; Lumite Co.)) and (b) “invertebrate access,” consisting of mesh with four large holes of approximately 30 cm by 30 cm, each (a 1.1 m × 1.5 m metal cage enclosed in mesh with large holes cut out of the mesh allowing access by invertebrates while controlling for potential effects of the mesh on light availability). These physical exclosures remained standing until the end of the study in 2015. Invertebrate reduction plots were additionally sprayed with a permethrin insecticide (Hi‐Yield Kill‐A‐Bug; Voluntary Purchasing Group, Bonham, TX, USA) to further reduce invertebrate abundance; this method has been shown to reduce invertebrate abundance by fourfold (Sanders et al., 2007). Insecticide was applied with a backpack sprayer at a rate of 0.23 L/m^2^ every two weeks throughout the growing season. For 6 weeks, we sampled the invertebrate community within the immediate area of the plots using sweep nets and sticky traps (similar to Lane (2006) and Sanders et al. (2007)). Invertebrate abundances did not differ between small mammal access and reduction areas. The most common invertebrates found at our site were red‐legged grasshoppers (*Melanoplus femurrubrum*), leafhoppers (*Cicadellidae*spp.), and little black ants (*Monomorium minimum*).

In a fully factorial design consisting of the previously described invertebrate manipulation plots, we manipulated soil N by adding 10 g/m^2^ of nitrogen in the form of urea pellets to half of the plots. Soil N manipulations began in July of 2013 and again in May of 2014 and 2015 following NutNet protocol (http://www.nutnet.umn.edu/) (Borer et al., 2013). This procedure mimics nitrogen deposition from agriculture and industrial sources in grasslands and old fields (Borer, Seabloom, Gruner et al., 2014a; Larson & Siemann, 1998; McLendon & Redente, 1992). We measured soil N by deploying ion‐exchange resin bags (H‐OH form, number R231‐500, Fisher Scientific International) approximately 5 cm below the soil surface in each plot in May of 2015. In August of 2015, we collected and air‐dried the bags. Resin beads were mixed with 2 mol/L KCl to extract NO_3_^−^ and NH_4_^+^ then later analyzed in solution with an autoanalyzer (Lachat Quikchem 8000, Hach) (Sanders et al., 2007). Analysis confirmed that across small mammal treatments, N values in the N addition plots were more than twice that of the control plots (NH_4_: *F*‐ratio 7.54, *p*‐value 0.003; total N: *F*‐ratio: 7.53, *p*‐value 0.003) (Supporting Information Appendix S3).

### 2.3 Field measurements

#### 2.3.1 Plant community

During the 2014 and 2015 growing seasons, we identified all plant species to determine richness (S) within the study plots and estimated species‐specific foliar cover in each experimental plot using modified Braun‐Blanquet cover classes with seven foliar cover categories (0%–2%, 2%–5%, 5%–15%, 15%–25%, 25%–50%, 50%–75%, 75%–100%) (Braun‐Blanquet, Fuller, & Conrad, 1932). We then used each foliar cover class median to represent species‐specific abundance. Next, we converted those data to relative abundance per species by dividing the species abundance by total abundance. We use the relative abundance data to calculate Shannon diversity index ($H^{{}^{\prime }}=-\sum_{i=1}^{S}J_{i}^{{}^{\prime }}\ln J_{i}^{{}^{\prime }}$) (Shannon & Weaver, 1949). We also calculated evenness (*J*′) as *H*′/ln(*S*)^2^. To determine the effects of herbivory and soil N on total community biomass (aboveground net primary productivity: ANPP), we clipped all individuals rooted in a 0.25 m^2^ area within each plot at ground level in fall of 2015. We oven‐dried the plant material at 65°C for approximately 48 hr and then weighed the samples to estimate ANPP.

#### 2.3.2 Microclimate

To determine how microclimate differed across herbivore and nutrient treatments, we measured light availability (photosynthetic photon flux density), soil moisture (volumetric water content, %), and temperature during both 2014 and 2015 growing seasons. We measured light availability and soil moisture at the beginning and peak of the growing season (May and August). To estimate light availability, we first removed the plot's cage then used a light‐integrating ceptometer (LP‐80 AccuPAR; Decagon Device, Inc.) to record and then average two measurements per plot. We used a handheld soil moisture probe (Hydro Sense II) to measure percent volumetric water content (%VWC) in two random spots in each plot and averaged within‐plot values. We recorded soil temperature by deploying iButtons (iButton^®^ Temperature Logger; Maxim Integrated; San Jose, CA) at the soil surface, tracking seasonal temperature fluctuations (May‐August).

### 2.4 Analyses

#### 2.4.1 Univariate analyses

Preliminary analyses showed that “Year” was a not a significant factor in our model. We therefore pooled our community structure data (richness, diversity, and evenness) and species‐specific foliar cover by averaging plot‐level values across the two‐year study (2014 and 2015). We performed a series of one‐way nested ANOVAs, one for each response variable. We analyzed the response of the plant community (aboveground biomass, richness, evenness, and diversity) and microhabitat (light availability, soil moisture, temperature, and soil N) to the herbivore and nutrient treatments using nested ANOVAs:

$$Y_{\mathrm{ijk}}=\mu +\text{Small}\;{\text{mammal}}_{2i}+\text{Soil}\;N_{j}{\left(\text{Small}\;\text{mammal}\right)}_{i}+{\text{Invertebrate}}_{j}\left(\text{Small}\;\text{mammal}\right)+{\text{c}}_{\mathrm{ijk}}$$

where μ is the overall mean, Small mammal is a main fixed effect, Soil N and Invertebrate are nested factors within Small mammal, and Є*_ijk_* is the residual error associated with the measured dependent variable *Y_ijk_*. We checked whether each response variable met normality assumptions and used Tukey's HSD as post hoc tests to test variability of nested combinations. We used JMP version 12 to analyze univariate data.

#### 2.4.2 Multivariate analyses

Similar to the univariate analyses, we used the pooled data for all multivariate analysis. We used a nonparametric, permutational multivariate analysis of variance (PERMANOVA) to determine the change in compositional similarity due to invertebrate herbivory and soil N in the context of small mammal herbivores (represented in our statistical model as the nested factors small mammals, invertebrates (small mammals), and N (small mammals)). We performed the PERMANOVA on a Bray–Curtis similarity matrix generated from the log‐transformed (log X + 1) plant composition data (i.e., foliar cover (N) explained above). A significant pseudo‐*F*‐ratio (the test static for the PERMANOVA) represents community compositional dissimilarity either due to separation of communities by treatment in multivariate space (also known as location) or due to variation of communities within treatments in multivariate space (also known as dispersion) (Anderson, 2001; Bunn, Jenkins, Brown, & Sanders, 2010). To determine whether compositional differences were due to location or dispersion differences, we followed PERMANOVA analyses with permutational multivariate analysis of dispersion (PERMDISP) analyses (Bunn et al., 2010). We used PRIMER version 6 (Plymouth Marine Laboratory, UK) for multivariate analyses.

To illustrate species composition in multivariate space, we performed a series of principal coordinate analyses (PCO) based on the Bray–Curtis similarity matrix. We used the first PCO axis, which accounted for a significant proportion of total variation in compositional similarities, to illustrate treatment differences in β diversity over time. We also performed a similarity percentage analysis (SIMPER) to determine which species contributed the most to overall differences in community composition dissimilarities between soil N and invertebrate herbivores in the context of small mammal herbivores.

## 3 RESULTS

### 3.1 Impact of mammal herbivory on plant community structure and function

**Figure 1:**
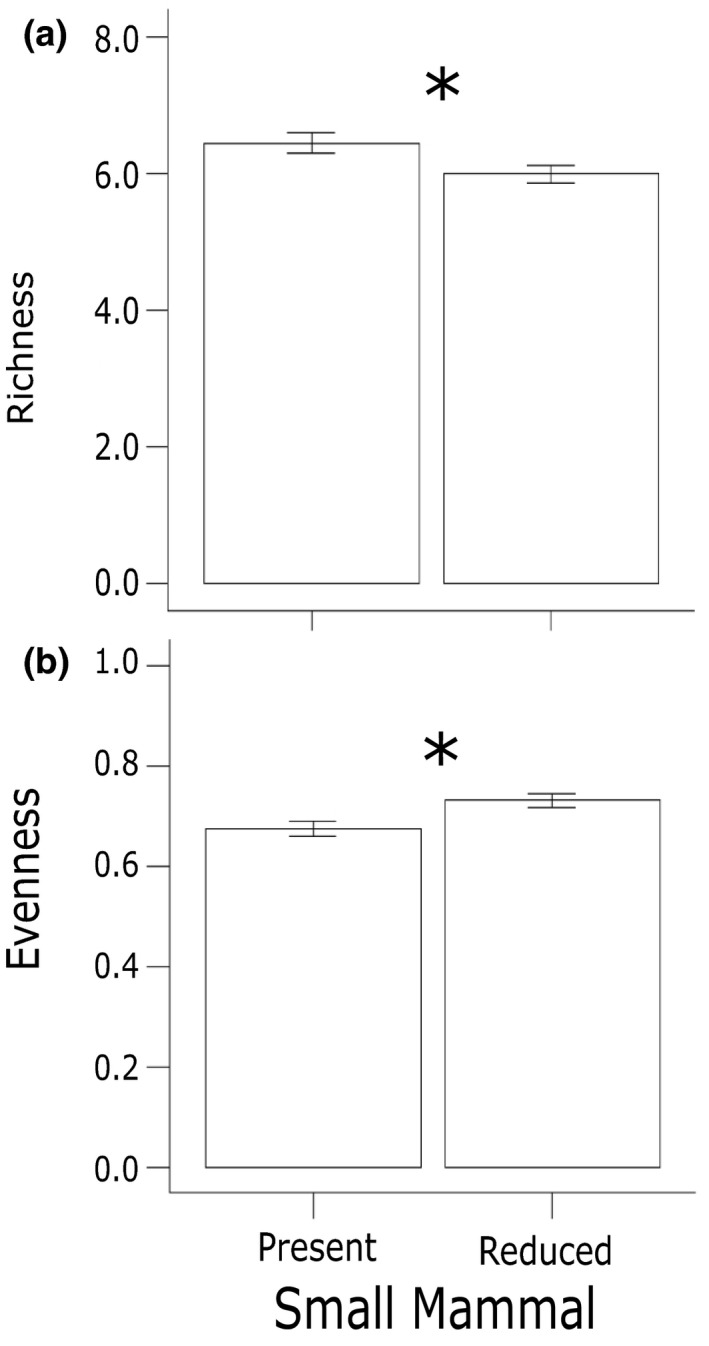
Small mammal reduction decreased species richness while increasing evenness. While small mammals were reduced, (a) species richness decreased by 7% (*p* = 0.002) and (b) evenness increased by 7% (*p* < 0.01)

**Figure 2:**
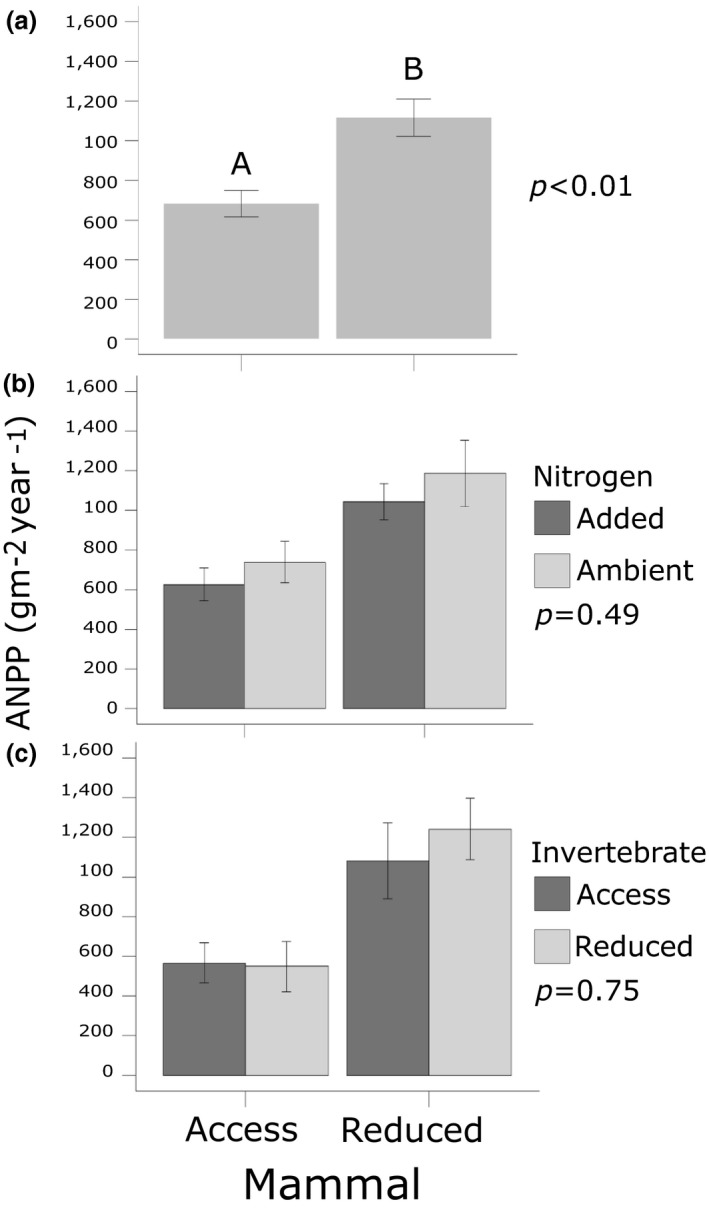
Small mammal herbivores decrease primary productivity. Mammals (a) decreased ANPP by more than half. Neither soil *N* (b) nor invertebrate herbivory (c) significantly altered ANPP. Bars with different letters denote significant differences. Error bars represent standard error

**Figure 3:**
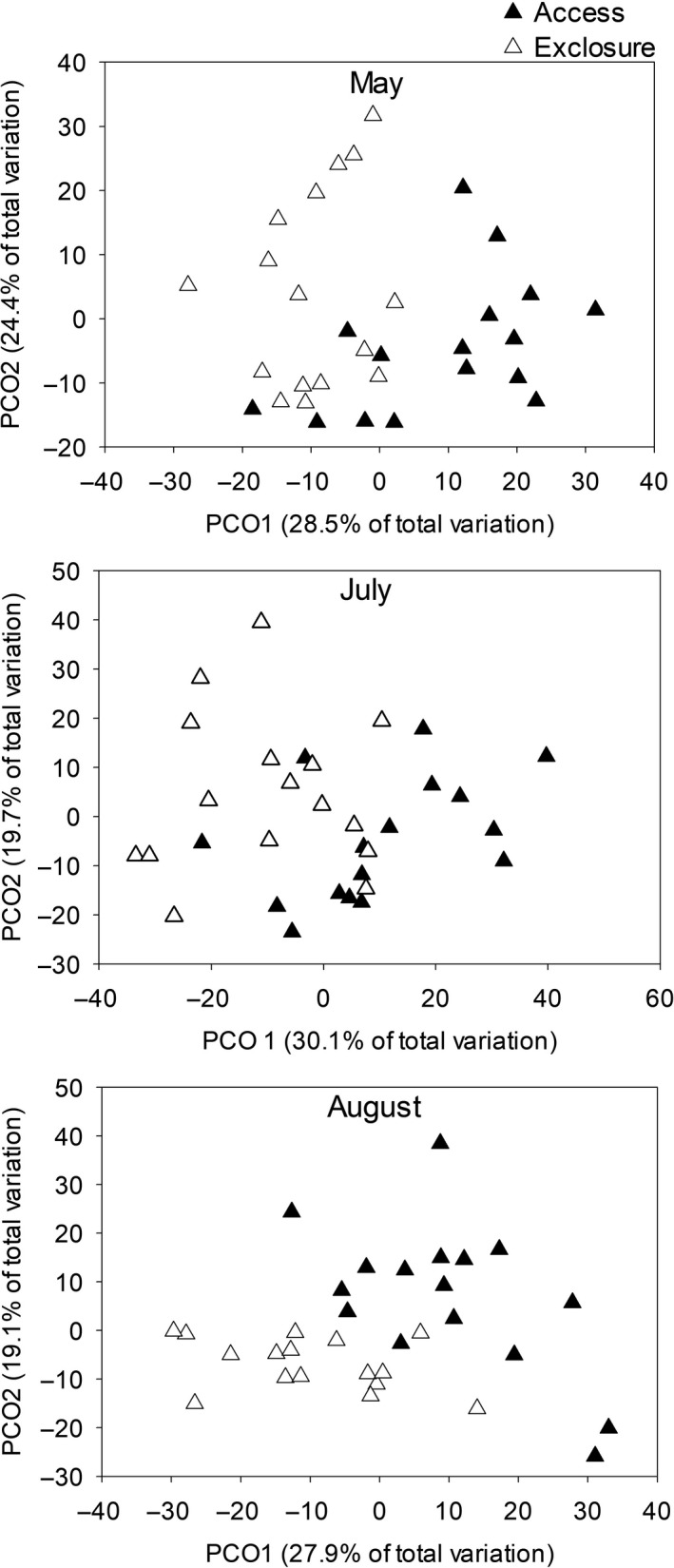
Small mammal herbivory leads to shifts in species composition (closed triangles = mammal access, open triangles = mammal reduction). Two‐dimensional representation of plant communities across two principal coordinate–ordinates

Small mammal reduction, compared to rodent access plots, lowered plant species richness yet promoted species evenness and aboveground biomass (Table 1). Species richness was 7% lower in small mammal reduction plots compared to access plots (*p* = 0.02) (Figure 1a). Unlike species richness, small mammal reduction led to a 7% increase in species evenness (*p* < 0.01) (Figure 1b). Additionally, total aboveground plant biomass was 50% greater in small mammal reduction plots compared to small mammal access plots (*p* < 0.001) (Figure 2a). Small mammals did not alter diversity, yet led to a decline in species compositional similarity (*p* = 0.02; Tables 2 and 3; Figure 3).

**Table 1:** Nested ANOVA results

Response	Source	*df*	*F*‐ratio	*p*‐value
Richness	Mammal	1	5.71	**0.02**
	*N*(mammal)	2	2.82	0.07
	Invertebrate (mammal)	4	0.65	0.63
Evenness	Mammal	1	9.83	**<0.01**
	*N*(mammal)	2	1.19	0.32
	Invertebrate (mammal)	4	3.96	**0.01**
Diversity	Small mammal	1	0.03	2.36
	*N*(Small mammal)	2	0.02	0.59
	Invertebrate (mammal)	4	0.16	2.73
ANPP	Mammal	1	14.47	**<0.001**
	*N*(mammal)	2	0.74	0.49
	Invertebrate (mammal)	4	0.29	0.75

Note

Bolded values represent statically significant values.

**Table 2:** PERMANOVA results based on composition

Source	*df*	Pseudo‐*F*	*p*‐value
Mammal	1	3.30	**0.02**
*N*(mammal)	2	2.62	0.33
Invertebrate (mammal)	2	1.65	0.11

Note

Bolded values are statically significant.

**Table 3:** PERMDISP results for plant species composition in multivariate space for each data collection time

Source	*df*	*t*	*p* (perm)
Mammal			
Acc,Exc	1,30	1.61	0.14
*N* (mammal)			
Acc[*N*,C]	3,28		
Exc[*N*,C]		1.05	0.33
Invertebrate (mammal)			
Acc[F,L]	3,28		
Exc[F,L]		1.23	0.35

Note

Letters represent different treatments: Acc = mammal access, Exc = rodent reduction, *N* = Nitrogen added, C = ambient *N*, *F* = full mesh (invertebrate reduction), L = leaky mesh (invertebrate access). Bolded values are statically significant.

### 3.2 Impact of nitrogen on plant community structure and function

**Figure 4:**
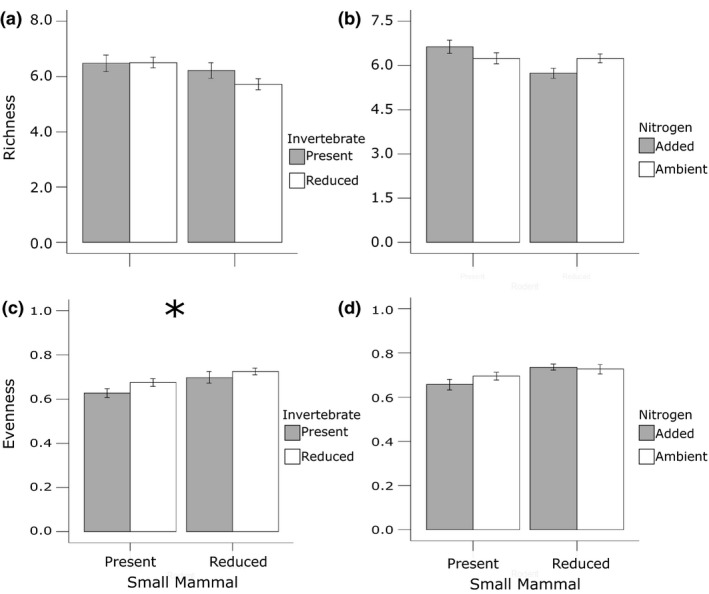
Invertebrate herbivory decreased evenness, yet N addition had negligible effects. Invertebrate herbivores (a) did not significantly alter richness (*p* = 0.63) yet (c) lowered evenness by 3.5% (*p* = 0.01). N addition did not significantly alter (b) richness (*p* = 0.07) nor (d) evenness (*p* = 0.32)

The effect of N addition on plant species richness and evenness was dependent on the reduction of small mammals. When small mammals were reduced, nitrogen addition lowered plant richness by 15% (*p* = 0.07) (Figure 4b). Nitrogen addition did not significantly alter diversity, evenness (Figure 4d), total aboveground biomass (Figure 2b), or species compositional similarity (*p* = 0.33) regardless of small mammal presence.

### 3.3 Impact of invertebrate herbivore reduction on plant community structure and function

The reduction of invertebrate herbivores altered species evenness, but these effects were dependent on small mammals. When small mammals were present, the reduction of invertebrate herbivores promoted evenness by 3.5% (*p* = 0.01; Figure 4c), but when small mammals were reduced, invertebrate herbivores had no effect on plant evenness. On the other hand, invertebrate herbivores did not significantly alter richness (*p* = 0.63; Figure 4a), diversity (*p* = 2.73), aboveground biomass (*p* = 0.75; Figure 2c), or compositional similarity (*p* = 0.11; Tables 2 and 3).

### 3.4 Microclimate responses to herbivores and nutrients

Herbivores and soil N altered abiotic conditions in our grassland ecosystem (Supporting Information Appendix S3). Small mammal reduction led to a decrease in light availability by 27% (*p* < 0.0001), but surprisingly did not influence soil temperature (*p* = 0.07) or soil moisture (*p* = 0.66). Insect reduction decreased mean soil temperature by 2% (*p* = 0.005), while not affecting light availability (*p* = 0.79) or soil moisture (*p* = 0.71) across small mammal treatments. Finally, soil N addition increased mean soil temperature by 6% (*p* = 0.01), but did not affect light availability (*p* = 0.27) or soil moisture (*p* = 0.19).

## 4 DISCUSSION

The reduction of small mammals lowered plant species richness and plant dominance while promoting aboveground biomass and altered the species compositional similarity of this focal temperate grassland plant community. Warm‐season grasses, which dominated in the reduction of small mammals, were replaced by perennial forbs when small mammals were present. Further, small mammals shaped the effects of soil N addition and invertebrate herbivore reduction on plant community structure. While the *reduction* of small mammals with soil N addition lowered plant species richness, the *presence* of small mammals with invertebrate reduction lowered plant dominance. Surprisingly, neither soil N addition nor invertebrate reduction altered plant diversity or productivity.

### 4.1 Vertebrate herbivores drive changes in plant community

Small mammal herbivores lowered plant productivity while promoting plant richness, leading to a decrease in plant species diversity. Our findings are in agreement with other studies showing that small mammal herbivores lower ANPP (Austrheim, Speed, Martinsen, Mulder, & Mysterud, 2014; Gruner et al., 2008; Olofsson, Tommervik, & Callaghan, 2012). However, our study of small mammals on plant community structure contrasts previously documented patterns. Previous studies showed evidence that selective feeding by small mammals altered species richness patterns of the plant community leading to a reduction of grassland diversity (Howe, Brown, Zorn‐Arnold, & Sullivan, 2001; Howe et al., 2006). Although we have also documented shifts in plant composition as a result of small mammal herbivory, plant diversity was ultimately promoted in our study. Overall, *A. gerardii* and *T. flavus* contributed most to the dissimilarly across small mammal treatments overtime; these species declined with small mammal herbivory. On the other hand, C_3_ forb species, such as *A. ludoviciana* and *A. psilostachya*, were more abundant in small mammal access plots than reduction.

Our data suggest that the decline of small mammal populations will promote C_4_ graminoid dominance in our system. This result conflicts with findings by Moorhead, Souza, Habeck, Lindroth, and Classen (2017) in which small mammal exclusion promoted C_3_ species rather than C_4_. Moorhead et al.'s study took place in a mesic grassland, likely favoring C_3_ perennial forbs rather than the C_4_ graminoids that are generally more tolerant of our xeric grassland system. With these contrasting conclusions, more studies are needed before generalizations can be made about the future plant community structure of grasslands.

### 4.2 Herbivore guilds have differing impacts on plant community responses

Herbivores of different guilds can have unique effects on plant community productivity and diversity (Bakker et al., 2006; Oduor et al., 2010; Shurin & Seabloom, 2005). Across guilds, differences in body size (Hopcraft, Olff, & Sinclair, 2010) and feeding preferences (Huntly, 1991) can lead to very different outcomes, primarily in plant diversity. Also, it is suggested that herbivory by small mammals leads to a greater relative change in total biomass than invertebrate herbivory (Hulme, 1996). Furthermore, herbivores can have different effects on plant diversity that are dependent on herbivore size (Liu et al., 2015). However, few studies have examined the variable effects of invertebrates and small mammal herbivores. Zhu et al. (2018) found that vertebrate herbivores selectively forage on high‐quality plant tissue, leaving behind low‐quality tissue and ultimately negatively impacting invertebrate herbivores and buffering their effects in Mongolian steppe meadows. Further, Liu et al. (2015) showed that differing herbivore guilds alter the ratio of forbs and grasses in ways that are contingent on background levels of plant diversity, ultimately driving complex shifts in biodiversity.

La Pierre et al. (2015) provide one of the few studies that, like ours, examines the interaction of invertebrate and vertebrate herbivores on terrestrial ecosystems. Unlike our study, La Pierre et al. (2015) found that invertebrates, rather than vertebrate herbivores, drove compositional shifts in a tallgrass prairie. La Pierre et al. (2015) explained that the shift in the plant species dominance, and composition specifically, was driven by an increase in the grass‐to‐forb ratio when invertebrates were present rather than removed. Although we also found invertebrate herbivores shape species evenness and diversity, such effects were mediated by small mammals. Different from La Pierre et al. (2015), invertebrate herbivores in our system promoted plant species dominance rather than shifting plant dominance patterns. Surprisingly, invertebrate herbivory in our system did not seem to drive species composition. But not surprisingly, and similar to other studies (Axelsson & Stenberg, 2012; La Pierre et al., 2015), invertebrate herbivory in our plots did not significantly affect total productivity. These studies suggest invertebrate herbivores differentially feed on plant material based upon litter quality. Therefore, the relative abundance of certain plants may change, but overall biomass remains the same.

While other studies have also failed to detect an effect of invertebrates on total productivity (La Pierre et al., 2015), this could also be evidence of a lag effect (Gruner et al., 2008; Howe et al., 2006; Souza et al., 2015). As mentioned in the methods, the small mammal plots were established approximately seven years prior to the invertebrate manipulation. It is possible that invertebrates must be reduced for longer than the two growing seasons in our study system to elicit plant productivity responses. However, it is important to reiterate the community diversity changes caused by the short‐term invertebrate manipulation observed over the two‐year duration of this study, suggesting this time frame is likely adequate for invertebrate impacts of be evident. Overall, our data show that different herbivore guilds can lead to unique independent and interactive effects on a plant community structure.

### 4.3 Soil N addition influences on plant community structure and function

We found that soil N addition lowered species richness similar to other studies (Clark & Tilman, 2008; Stevens et al., 2010; Suding et al., 2005). Interestingly, we found this effect to be true only when small mammals were reduced. Increased nutrient availability favors competitive dominance and exclusion of rare species (Hillebrand, Bennett, & Cadotte, 2008; Stevens, Dise, Mountford, & Gowing, 2004) leading to a decrease in the total number of species present. The loss of species is especially apparent when small mammal herbivores are not mediating such effects, with the presence of small mammals counteracting such effects.

Surprisingly, soil N addition had very little effect on diversity and evenness, regardless of vertebrate herbivore presence. However, other studies in similar systems have also shown that herbivores (across guilds) and fertilization do not have interactive effects on plant productivity and diversity (Blue, Souza, Classen, Schweitzer, & Sanders, 2011; Gruner et al., 2008; Souza et al., 2015). It is possible that N is not the limiting nutrient in our system; instead, another nutrient, such as phosphorus, may be limiting productivity here (Blue et al., 2011).

Soil N addition did not significantly alter the microclimate. Herbivory and eutrophication have conflicting effects on plant community productivity and diversity. However, herbivory may mediate the effects of eutrophication by alleviating light limitation. Borer, Seabloom, Gruner et al. (2014a) suggest that an increase in ground‐level light should correspond to a decrease in productivity and increase in diversity. In the context of herbivores and nutrients, they propose that an increase in ground‐level light by herbivory can counteract the effects of eutrophication from fertilization to influence plant diversity. Our study shows soil N addition does not alter light availability in either the presence or the absence of small mammals. Without such an impact on the microclimate, soil N does not counteract the effects of the herbivores in our study.

## 5 CONCLUSION

We found that small mammal herbivores drove overall plant diversity, compositional similarity, and plant productivity, but also played a key role in mediating the effects of invertebrate herbivores and soil N on plant dominance and richness, respectively. Small mammal reduction promoted both plant productivity and species compositional dissimilarity while simultaneously lowering plant richness and plant dominance. Further, small mammals mediated the effects of soil nitrogen and invertebrates on species richness and evenness, respectively, yet in different directions. Specifically, soil N effects on plant richness were mediated by the presence of small mammals, while the impacts of invertebrate herbivory on plant evenness were contingent on small mammal absence. Surprisingly, herbivores and nutrients had little impact on microclimate, but it is important to note the coarse temporal resolution that we tracked abiotic variables.

These data add to the recently established paradigm of top‐down control on primary productivity and diversity in terrestrial systems (Loreau et al., 2001) and also suggest that these top‐down controls may mediate bottom‐up effects (Schmitz, 2003; Schmitz, Hamback, & Beckerman, 2000). Further, there appear to be strong cross‐guild contingencies driving the impacts of different herbivore guilds on plant communities, ultimately driving plant functional group composition. In future studies, we suggest it is imperative to consider herbivore guild type along with the interactions between these herbivores and resource availability.

## AUTHOR CONTRIBUTION

KS and LS designed research; KS, LS, NP performed the research; KS, LS, NP processed, summarized, and analyzed the data; KS, LS, NP wrote the paper.

## Data Availability

Plant community and microclimate data can be accessed in the Dryad Digital Repository https://doi.org/10.5061/dryad.qc042n4.
