# Supplementary material for: Small mammal herbivores mediate the effects of soil nitrogen and invertebrate herbivores on grassland diversity
Source: Ecol Evol. 2019 Feb 21;9(6):3577–87. doi: 10.1002/ece3.4991 (PMC6434553; doi:10.1002/ece3.4991)
Supplement: Supplementary file 1 [file ECE3-9-3577-s001.docx]

**Appendix 1.** Arrangement of experimental plots in the field. Each circle is a plot with the symbol representative of the N treatment (+ = N added; - = ambient N) and the letter representative of the invertebrate treatment (F=full mesh, invertebrate reduction; L=leaky mesh, invertebrate access). Solid lines represent fences. N and invertebrate herbivore treatments were randomly assigned to plots within the mammal access and the mammal reduction areas.

**
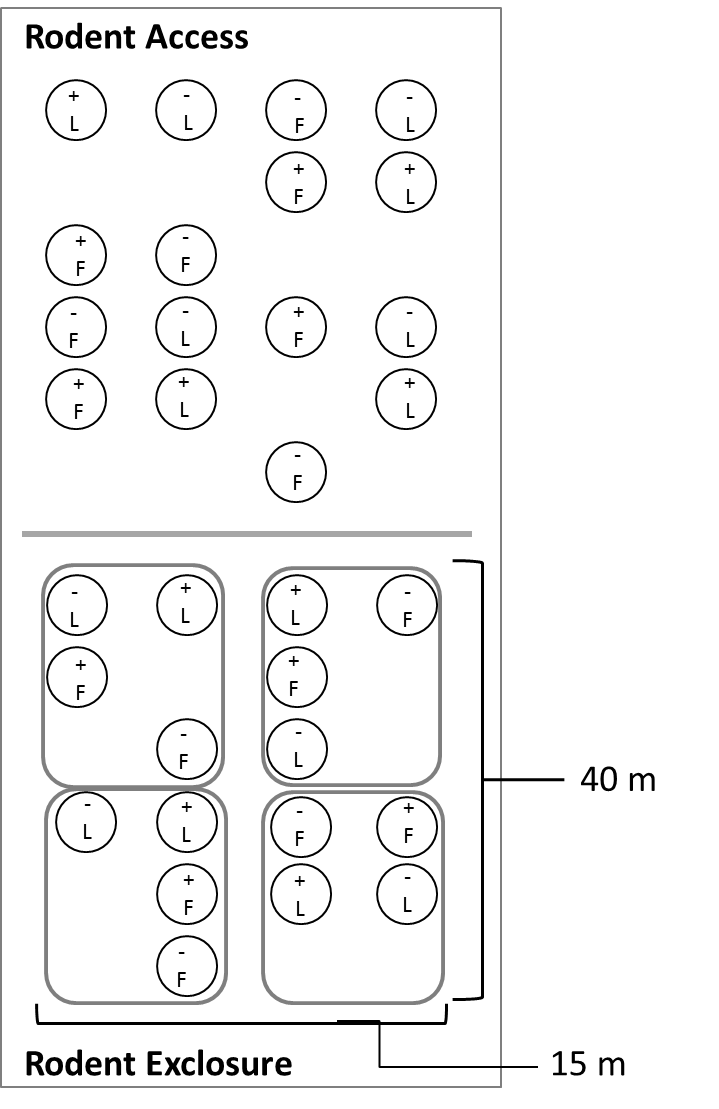
**
