# Supplementary material for: Small mammal herbivores mediate the effects of soil nitrogen and invertebrate herbivores on grassland diversity
Source: Ecol Evol. 2019 Feb 21;9(6):3577–87. doi: 10.1002/ece3.4991 (PMC6434553; doi:10.1002/ece3.4991)
Supplement: Supplementary file 2 [file ECE3-9-3577-s002.docx]

| **Day** | **Plot** | **Total Number** | **# Traps** | **Standardized Density** |
| --- | --- | --- | --- | --- |
| 1 | Access | 8 | 15 | 0.53 |
| 1 | Exclosure | 7 | 70 | 0.10 |
| 2 | Access | 3 | 30 | 0.10 |
| 2 | Exclosure | 2 | 60 | 0.03 |
| 3 | Access | 12 | 30 | 0.40 |
| 3 | Exclosure | 9 | 60 | 0.15 |

**Appendix 2.** Small mammal abundance data from three consecutive dates of trapping (“Day”) for small mammal “Access” plots and “Exclosure” plots, along with the total number of traps and standardized density (see Table below). Small mammal composition from trapping data for “Rodent Access” vs. “Rodent Exclosure” plots (Figure below).
