# Supplementary material for: Small mammal herbivores mediate the effects of soil nitrogen and invertebrate herbivores on grassland diversity
Source: Ecol Evol. 2019 Feb 21;9(6):3577–87. doi: 10.1002/ece3.4991 (PMC6434553; doi:10.1002/ece3.4991)
Supplement: Supplementary file 3 [file ECE3-9-3577-s003.docx]

**Appendix 3.** Microhabitat. Shown are mean values and p-values. Bolded p-values are significant (p<0.05). Letters represent values significantly different from one another.

| **Microhabitat** | | | | | | | |
| --- | --- | --- | --- | --- | --- | --- | --- |
|  |  | Soil Moisture | Temperature | Light Availability | Soil Ammonium | Soil Nitrate | Total Soil N |
| Access |  | 13.0 | 26.5 | 854.3 ^A^ | 53.0 | 0.3 | 46.7 |
| Reduction |  | 12.2 | 26.5 | 626.0 ^B^ | 74.5 | 0.3 | 65.4 |
| p-value | | 0.1 | 0.9 | **0.0** | 0.1 | 0.3 | 0.10 |
| Access | N | 13.7 | 26.2 ^A^ | 832.2 | 31.9 ^AB^ | 0.3 | 24.2 ^AB^ |
|  | C | 12.4 | 26.9 ^B^ | 876.4 | 68.8 ^B^ | 0.3 | 69.1 ^B^ |
| Reduction | N | 12.5 | 26.8 ^B^ | 567.9 | 35.8 ^A^ | 0.3 | 31.6 ^A^ |
|  | C | 11.9 | 26.1 ^AB^ | 684.0 | 113.2 ^B^ | 0.3 | 99.3 ^B^ |
| p-value | | 0.2 | **<.01** | 0.5 | **<.01** | 0.2 | **<.01** |
| Access | L | 13.3 | 26.6 ^AB^ | 834.1 | 45.7 | 0.3 | 40.2 |
|  | F | 12.8 | 26.4 ^AB^ | 874.5 | 60.4 | 0.3 | 53.1 |
| Reduction | L | 12.3 | 27.0 ^A^ | 629.9 | 63.1 | 0.3 | 55.5 |
|  | F | 12.1 | 26.0 ^B^ | 622.0 | 85.8 | 0.3 | 75.4 |
| p-value | | 0.7 | **<.01** | 0.9 | 0.70 | 0.6 | 0.70 |
